# The Validation of Beck’s Depression Inventory in Patients With Systemic Diseases: A Psychometric Study at a Dental Institute

**DOI:** 10.7759/cureus.49830

**Published:** 2023-12-02

**Authors:** Visalachi MR, Roland Prethipa P

**Affiliations:** 1 Department of Oral Medicine and Radiology, Saveetha Dental College and Hospitals, Saveetha Institute of Medical and Technical Sciences, Saveetha University, Chennai, IND

**Keywords:** psychiatry and mental health, systemic diseases, special care dentistry, screening tools, depression

## Abstract

Background

Depression seriously threatens the world's public health, manifesting symptoms like loss of interest, fatigue, guilt, and impaired focus. Major depressive disorder is a common problem for those with chronic systemic illnesses. Since this illness has an impact on psychosocial well-being and interacts with anxiety and stress, it is crucial to assess psychological functioning. Depression-related issue has a negative impact on oral health and can cause cognitive dysfunction, social interaction problems, and low self-esteem. For the purpose of identifying and effectively managing depression in patients with systemic illnesses undergoing dental procedures, screening tools like the Beck's Depression Inventory (BDI) were used. The BDI's benefits include its strong internal consistency, sensitivity to change, broad concept validity, adaptable criteria validity for separating depressed and nondepressed people, and global dissemination.

Materials and methods

A cross-sectional prospective study was carried out after receiving the necessary institutional ethical approvals, and the participant's agreement was also obtained. The questionnaire was explained to the patients. The entire participation was voluntary.

Results

In the current study patient’s ages ranged from 34 years to maximum age of 83 years with a mean age of 59.70±13.16 years, with 40% of the population falling into the 51-65 years age group. According to the results, participants who had multiple systemic disorders were more likely to experience depression than people who had one systemic illness. The chi-square test showed no statistically significant results between the depression scores and the number of systemic diseases (p-0.574). Seventy-five per cent of individuals with depression required tooth extractions.

Conclusion

Those with systemic disorders who were receiving dental care in the current study showed elevated levels of depression. An important finding was the correlation between depression and tooth extraction, highlighting the need for proactive mental health assessments in dental care. Tailored interventions can mitigate the impact, enhancing the holistic well-being of patients suffering from systemic diseases and depression.

## Introduction

In the realm of dental care, the intricate relationship between mental health, systemic diseases, and their impact on oral well-being takes centre stage. Depression, a prevalent concern, casts a significant shadow on patients navigating both dental procedures and systemic disorders. An important global public health concern is depression, a common mental condition marked by reduced enjoyment and enthusiasm, decreased energy levels, emotions of guilt or diminished self-esteem, disrupted sleep or appetite patterns, and compromised ability to focus [1]. Systemic disorders are medical conditions that impact one or multiple systems within the body, such as the respiratory, immune, neurological, circulatory, or digestive systems. These disorders can evolve over time and frequently remain concealed or not readily apparent [2]. Individuals dealing with chronic medical conditions experience a notable prevalence of major depressive disorder [3]. The psychosocial well-being of individuals is impacted by depression, anxiety, and stress so it's important to examine psychosocial functioning in understanding mental health [4]. The presence of depression can have wide-ranging effects on a patient's oral well-being. It can lead to detrimental impacts like negative cognitive patterns, disruptions in social engagements and work productivity, and a decline in self-assurance and self-assuredness, all of which collectively influence the individual's overall quality of life (QoL) [5]. Individuals grappling with depression tend to exhibit higher incidences of dental caries, bad breath, gingivitis, and periodontitis [6]. While clinical diagnosis remains the gold standard for identifying depression, the use of screening methods is essential for estimating the prevalence of depression in epidemiological research and aiding in the screening, diagnosis, and treatment monitoring within clinical settings. Screening tools hold particular value in community surveys and in guiding patients toward psychiatric care [7]. Rating scales serve as research tools capable of methodically validating clinical judgments, decision-making processes, and psychopathological hypotheses. Additionally, they function as a measurement tool converting both implicit and explicit observations into quantitative data [8]. In order to assess depression, a number of scales are used, including the Beck's Depression Inventory (BDI-I, II), Zung Self-Rated Depression Scale, Center for Epidemiologic Studies Depression Scale, WHO (Five) Well-Being Index, Hospital Anxiety and Depression Scale (HADS), and Hamilton Depression Rating Scale. The BDI, which Beck et al. initially developed and validated in 1961, is the scale that is most frequently used among them [7]. Utilizing the BDI, this study explores a more nuanced understanding in an effort to open the door for more focused and successful dental care interventions for this vulnerable population. The current study aimed to assess the levels of depression using a BDI-I questionnaire, and delves into the dynamics of depression within a dental institute setting, emphasizing its profound implications on the mental and oral health of individuals contending with systemic diseases.

## Materials and methods

Study design

A cross-sectional prospective descriptive study was carried out using a BDI-I questionnaire, among (N=20) patients. In order to prepare for a more extensive study with a larger data set, this pilot study was designed in such a way as to evaluate research methodology, the viability and feasibility of data collection, potential challenges as well as difficulties faced. The research further looked into whether systemic disease patients who were receiving dental care exhibited higher rates of depression than those who weren't.

Inclusion and exclusion criteria

The study encompassed individuals with systemic illnesses who expressed their willingness to participate. All genders were eligible, and the participants were 18 years to 70 years and above, proficient in reading and completing the questionnaire in English. The study excluded individuals who were unwilling to participate, those with unclear or unknown systemic conditions, individuals diagnosed with a systemic condition but not on regular medication, and special care patients or those unable to provide consent independently.

Data collection

The study was conducted in the Special Care Dentistry Unit of the Department of Oral Medicine and Radiology. The study was carried out after receiving the necessary institutional ethical approvals (Ethical Clearance Number: IHEC/SDC/OMED-2104/23/201) and after getting the participants' agreement. The study and questionnaire were explained to the patients. The entire participation was voluntary. Patients were instructed to complete the BDI-I.

Statistical analysis

The collected data was tabulated and entered into the Excel worksheet (Microsoft® Corp., Redmond, WA). Results were obtained using the Statistical Package for the Social Sciences (IBM SPSS Statistics for Windows, IBM Corp., Version 23.0, Armonk, NY) and descriptive analysis was performed to analyze the age and gender and a p-value of 0.05 was set for statistical significance.

BDI scale

**Figure 1:**
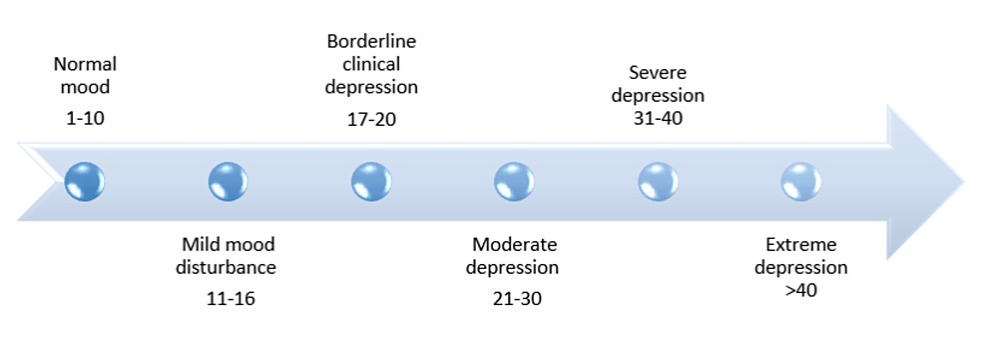
Scores indicating the levels of depression The figure depicts the categorization of depression levels based on the Beck’s Depression Inventory (BDI) questionnaire scores. A score of 1-10 denotes normal mood, 11-16 mild, 17-20 moderate depression, 31-40 severe and more than 40 indicating extreme level of depression.

The BDI scale, developed and approved by Beck et al. in 1961, served as the methodology for the current investigation. This evaluation instrument was intended to measure the level of depression in people 13 years of age and older to identify the disorder's severity. The Likert scale was used to give each of the 21 items, which make up the survey, a score between 0 and 3. This inventory comprised a series of statements reflecting various emotional and behavioural facets associated with depression. The higher the score, the more pronounced the level of depression. Patients were categorized based on their obtained scores. BDI can serve as a self-assessment instrument, empowering patients to take an active role in their mental health care and providing a structured avenue to express their emotions and experiences (Figure 1).

## Results

**Figure 2:**
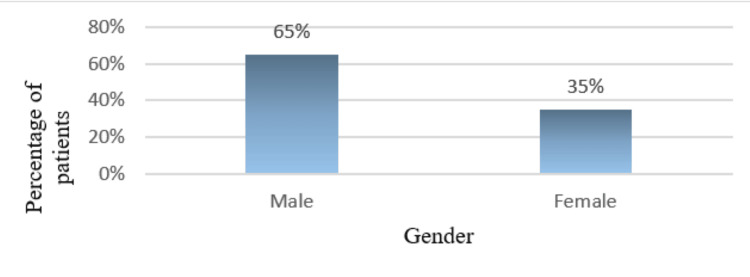
The figure depicts the overall distribution of gender among the study participants with the tendency for male predisposition (65%).

**Figure 3:**
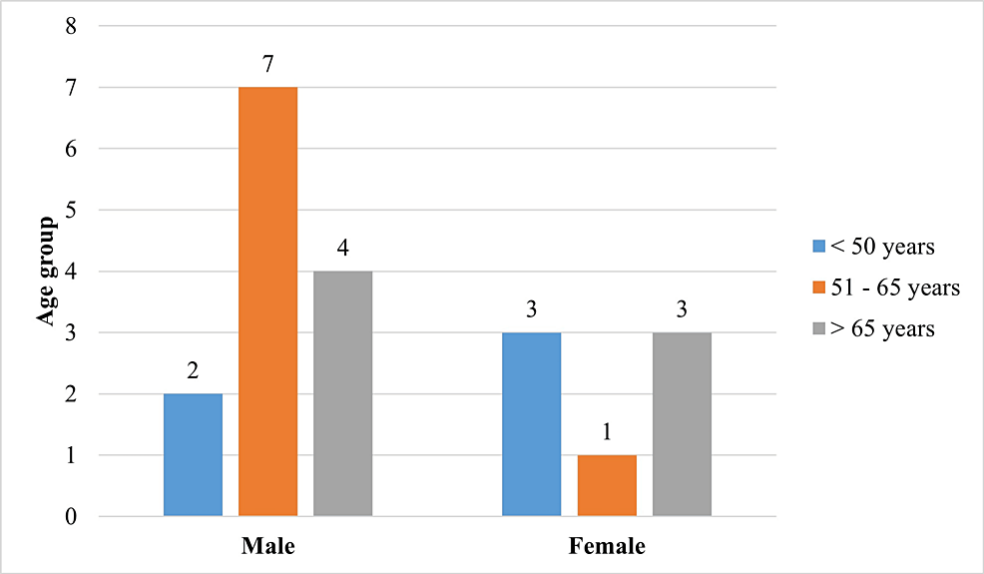
The figure shows the distribution of age with the majority of patients falling under the age group of 51-65 years.

**Figure 4:**
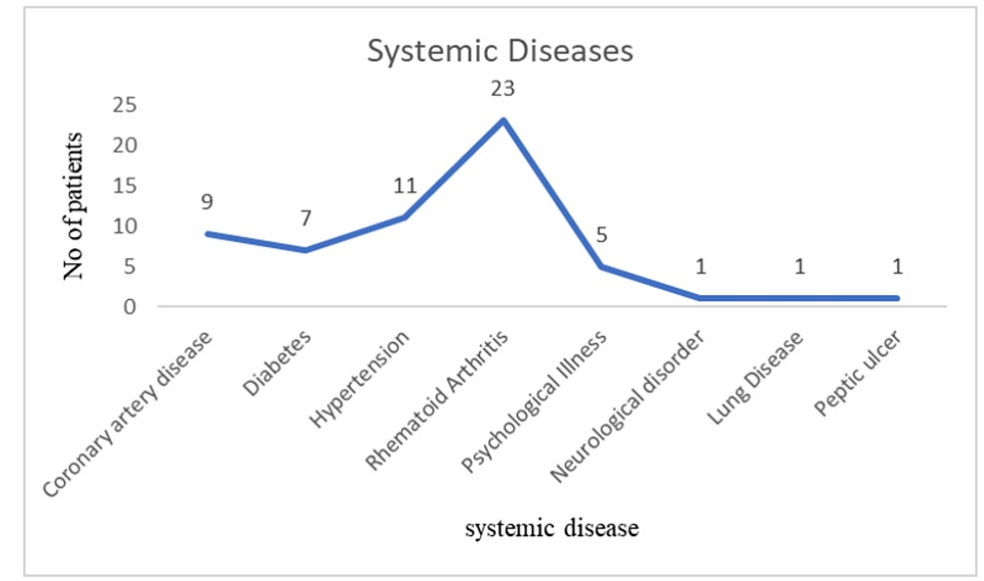
The figure depicts the existence of various systemic diseases among the study participants, where rheumatoid arthritis was the most prevalent, followed by hypertension, coronary artery disease and diabetes.

Figure 2 shows the distribution of gender among the study participants with a male preponderance of 65% and females 35%. The minimum age of the study population is 34 years, and the maximum age is 83 years. The mean age is 59.70±13.16 years. Figure 3 depicts the age range of the patients with the majority of the participants in the age range of 51 to 65 years. Age and gender demonstrated statistical significance with p value of 0.001. Figure 4 elucidates the presence of systemic co-morbid among the study participants with 23 participants diagnosed with rheumatoid arthritis, 11 hypertensive, nine coronary artery disease and seven diabetic patients. Table 1 depicts the extent of depression severity among individuals undergoing dental treatment where a considerable number of individuals were under the category of extreme depression (p-0.715). Table 2 offers a visualization of the correlation with the multiple co-existing systemic illnesses with more than three (10%), three systemic illnesses (20%), two systemic conditions (40%), with only one systemic disorder (30%) participants (p-0.574).

**Table 1:** Correlation between dental procedures and depression scores p-value > 0.05, not significant, Chi-square test

Dental treatment	Depression score					p-value
	Normal	Mild depression	Clinical depression	Moderate depression	Severe depression	
Extraction	1 (5)	3(15)	4 (20)	2 (10)	5 (25)	0.715
Scaling	0	0	0	0	1 (5)	
Prosthesis	0	0	0	0	3 (15)	
Restoration	0	0	1 (5)	0	0	

**Table 2:** Correlation between depression score and co-existing systemic disease p-value > 0.05, not significant, Chi-square test

Systemic disease	Depression score					p-value
	Normal	Mild depression	Clinical depression	Moderate depression	Severe depression	
One systemic disease	0	0	2 (10)	2 (10)	2 (10)	0.574
Two systemic diseases	1 (5)	2 (10)	2 (10	0	3 (15)	
Three systemic diseases	0	1 (5)	1 (5)	0	2 (10)	
More than three systemic diseases	0	0	0	0	2 (10)	

## Discussion

The primary factor in disability and a major contributor to the burden of disease globally is depression. It's important to stress that patients with systemic diseases are more prone to develop depression or symptoms related to it than the general population [9]. The BDI, which consists of 21 items, is a self-assessment tool created to examine the significant aspects of depression. These attributes encompass emotions, negative outlook, sensations of inadequacy, discontent with oneself, self-censure, contemplation of suicide, tearfulness, social withdrawal, challenges in decision-making, shifts in self-perception, work-related struggles, sleep disturbances, tiredness, reduced hunger, weight loss, excessive focus on physical symptoms, and diminished sexual drive [10]. Although it was initially developed to assess the intensity of depression symptoms in people with mental illness, it is now widely used as a diagnostic tool for determining depression in clinical settings and research [11].

The prevalence of depression is 13.4% in Virginia, USA, and 22.5% in Pakistan [12], and depression among Indians was highest with Jharkhand population 4.52%, followed by Tamil Nadu, West Bengal, Manipur, Rajasthan, Kerala, Uttar Pradesh, Punjab, Chhattisgarh, Madhya Pradesh, Assam, and Gujarat being the least with 1.01% [13]. A study by Seifu B et al. [14] stated that women suffer from depression more when compared to males. This variation in depression prevalence is related to elements like the societal power disparity that women suffer and the distinctive gender role indoctrination that pushes women to communicate their fears and anxieties in public. According to a study by Angst J et al. [15] males experience undiagnosed depression at considerably higher rates than females.

According to the results of the current study, those who have more than two systemic disorders are more likely to experience depression than people who only have one systemic comorbidity. Similarly, Joshipura et al. stated that chronic illnesses like oral cancer, diabetes, hypertension, and heart disorders have a propensity to make people with dental disease more vulnerable, which raises their level of depression [16]. In addition to other chronic conditions, depression frequently co-occurs with other chronic illnesses such as thyroid disease, diabetes, and heart issues. No matter what other factors may be at play, this coexistence has the ability to predict systemic illness and total mortality on its own. Unfortunately, at the moment, a sizable number of older people suffering from depression brought on by these medical illnesses go unreported in clinical settings because they lack support from their families and the community [17].

Maintaining optimal oral hygiene is of utmost importance, as its implications extend beyond mere dental and oral well-being. It significantly influences our overall health, physical appearance, and self-esteem. Contemporary studies have underscored a direct correlation between significant health issues such as diabetes, cardiovascular disorders, respiratory ailments, renal disease, and irregularities in dental health [18]. Similarly, a study by Kisely et al. [19] identified a link between severe depression and dental caries, attributing them to lifestyle modifications and access to dental care, while a study by Elter et al. [20] in the United States found a link between those with depression and severe periodontal disease and stated depressive medications can lead to xerostomia and periodontal inflammation. Numerous depressive symptoms have the potential to make it difficult for people to maintain good dental hygiene. It can be detrimental to have symptoms including drowsiness, psychomotor slowness, and feelings of inadequacy. Pain is frequently intertwined with depression and related mental conditions. There is an association between depression and temporomandibular joint pain, according to the study reported by Vimpari et al. [21]. The study by Anttila et al. [22] identified an association between depressive symptoms and oral dryness as well as decreased frequency of tooth brushing, regularity of dental visits, and increased lactobacillus counts, which in turn increases the susceptibility to oral diseases.

According to the results of this study, teeth extractions were required for 75% of individuals who had depression. The study also unveiled that comparing those who had scaling to those who had tooth extractions, depression was more than three times more common in the latter group. Correspondingly, results from a study conducted in the USA exhibited a similar trend, with tooth removal being associated with a threefold increased likelihood of developing depression [23]. The observed outcomes in this study could potentially be attributed to the distressing and uncomfortable nature of tooth extraction experienced by the participants. Instead, those who underwent tooth extraction may have been influenced by elements such as local anaesthesia, treatment outcomes, and the precise location of the extraction.

Understanding the underlying causes of depression is essential to effectively treat depressed patients because it can have many different manifestations, including specific issues like gag reflex, blood aversion, loss of sensation, poor pain tolerance, and fear of dentists as a result of traumatic past dental experiences. So, effective communication skills and rapport with patients are crucial. Treatment approaches for depressed patients often involve systematic desensitization, gradually exposing individuals to feared stimuli while encouraging relaxation strategies. As patients accumulate positive experiences, the fear tends to diminish.

The QoL can be significantly compromised by depression, leading to heightened reliance on external support. If not addressed, this condition can result in substantial clinical and societal ramifications. Timely identification, diagnosis, and prompt intervention including the initiation of treatment and rehabilitation hold the potential to avert distress, and untimely mortality, and foster a self-sufficient and constructive lifestyle. Swift recognition and proactive measures can likewise markedly diminish suicide rates, and mortality linked to medical ailments, and curtail healthcare expenses [9].

As per the findings of Sadi et al. [24], dental depression and stress are prevalent occurrences and are significant factors causing numerous individuals to avoid dental treatment. Beyond impacting the individual's overall QoL, depression also complicates the provision of care for these patients [25,5]. A person's QoL is impacted by depression. The assessment of a person's goals, standards, and concerns in relation to the society and value systems they belong to is referred to as their QoL [26]. This broad concept includes one's physical health, mental attitude, amount of independence, social connections, and connection to even the smallest components of their environment. If a person has a major mental disease, their QoL, which is associated with their health, is more likely to decline [27]. Nonetheless, there is universal agreement that effective treatment of depression should go beyond simply lowering symptom severity to also involve lessening functional limitations and restoring health-related QoL [28]. Regularly incorporating comprehensive health assessments into healthcare delivery is crucial. These assessments enable us to quantitatively measure patient's expectations and their level of satisfaction with the treatment rendered [29].

Evaluation of a wide range of dental diseases as well as oral health-related QoL was not assessed in the study, and it was not possible to analyze every oral disease and systemic disease separately. An increased risk of psychological illnesses, especially depression, has been associated with a number of systemic diseases. For individuals struggling with these complex medical concerns, comprehensive care must include understanding and treating depression as depression has been linked to worsening systemic illness outcomes, decreased self-care, and non-adherence to medical interventions. So, identifying and treating depression can benefit the patient's general health and well-being. So, future research will be directed with a larger sample size, examining the relationship between each systemic disease and the severity of depression as well as the patient's oral health and QoL.

## Conclusions

In the current study, patients with systemic disorders who were receiving dental care showed elevated levels of depression. An elevated level of depression was found to significantly correlate with tooth extraction. BDI tool facilitates better evaluation of mental health among patients receiving dental treatment with systemic diseases. The findings highlight the interconnectedness of psychosocial well-being, oral health, and overall QoL in this patient population. Addressing depression is imperative for enhancing treatment outcomes and promoting a holistic approach to healthcare.

## Figures and Tables

**Figure 5:**
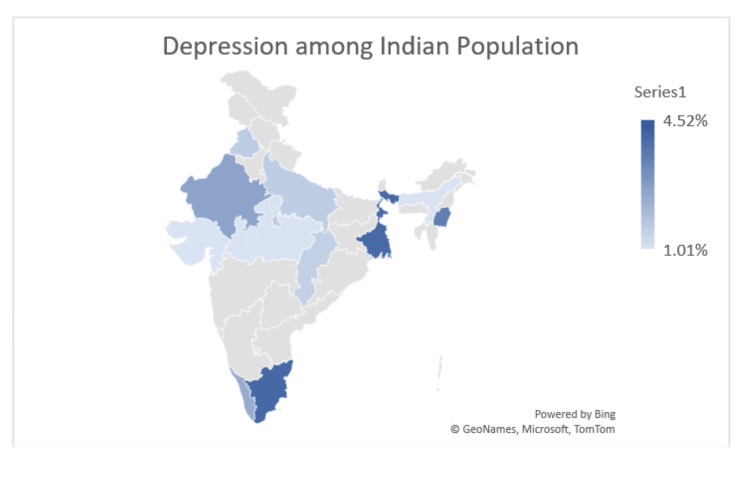
The figure illustrates depression among the Indian population according to the National Mental Health Survey of India 2015-2016. A darker shade of blue indicates a higher prevalence whereas a lighter shade denotes a decreased prevalence. Ref: [13] Image Courtesy: Visalachi MR, Roland Prethipa P

## Appendices

The link for Beck's Depression Inventory-I survey: https://www.ismanet.org/doctoryourspirit/pdfs/Beck-Depression-Inventory-BDI.pdf
